# Antidepressive Effects of Kamishoyosan through 5-HT1AReceptor and PKA-CREB-BDNF Signaling in the Hippocampus in Postmenopausal Depression-Model Mice

**DOI:** 10.1155/2019/9475384

**Published:** 2019-11-03

**Authors:** Shoko Shimizu, Yugo Ishino, Takashi Takeda, Masaya Tohyama, Shingo Miyata

**Affiliations:** ^1^Division of Molecular Brain Science, Research Institute of Traditional Asian Medicine, Kindai University, Osaka-Sayama, Osaka 589-8511, Japan; ^2^Division of Women Medicine, Research Institute of Traditional Asian Medicine, Kindai University, Osaka-Sayama, Osaka 589-8511, Japan; ^3^Osaka Prefectural Hospital Organization, Osaka, Osaka 541-8567, Japan

## Abstract

Females are well known to suffer disproportionately more than males from stress-related neuropsychiatric disorders, especially during perimenopausal and postmenopausal periods. In addition to a decline in serum estradiol levels, environmental stress and social stress likely contribute to the development of neuropsychiatric symptoms in perimenopausal and postmenopausal women. Kamishoyosan (KSS) is a traditional Japanese Kampo medicine, composed of a specified mixture of 10 crude compounds derived from plant sources, widely used for various neuropsychiatric symptoms in perimenopausal and postmenopausal women. However, the molecular mechanisms underlying KSS-mediated attenuation of neuropsychological symptoms and stress-response behaviors in perimenopausal and postmenopausal women remain unknown. In the present study, we first established a mouse model for postmenopausal depression-like signs using chronic water-immersion and restraint-stressed ovariectomized (OVX) mice to investigate the underlying molecular mechanism of KSS. We found that continuous administration of KSS to these mice normalized the activation of the hypothalamic-pituitary-adrenal (HPA) axis, ameliorated stress-induced depressive behavior, and prevented a decrease of neurogenesis in the hippocampus. As previous studies have implicated dysfunction of the hippocampal 5-HT1A receptor (5-HT1AR) in depressive disorders, we also evaluated the effect of KSS on 5-HT1AR expression and the protein kinase A- (PKA-) cAMP response element-binding- (CREB-) brain-derived neurotrophic factor (BDNF) signaling pathway in the hippocampus in this model. The level of 5-HT1AR in the hippocampus decreased in chronic stress-exposed OVX mice, while KSS treatment normalized the stress-induced decrease in 5-HT1AR expression in the hippocampus of chronic stress-exposed OVX mice. Furthermore, we found that KSS treatment upregulated the expression levels of phosphorylated PKA (p-PKA), phosphorylated CREB (p-CREB), and BDNF in the hippocampus in chronic stress-exposed OVX mice. These results suggest that KSS improves neuropsychiatric symptoms through 5-HT1AR and PKA-CREB-BDNF signaling in the hippocampus in postmenopausal women.

## 1. Introduction

Depressive disorders are probably the oldest and the most frequently diagnosed psychiatric illnesses and thus are classified as common mental disorders (CMDs) [1, 2]. Depressive disorders are characterized by emotional, cognitive, autonomic, and endocrine function disturbances, affecting approximately 10–20% of the global population in any given year [1–3].

Females are well known to be diagnosed with CMDs disproportionately more than males, especially during the perimenopausal and postmenopausal periods [4–6]. It is also well known that menopausal symptoms are linked to decreased serum estradiol levels, which mediate a variety of physical and psychiatric signs and symptoms [7, 8]. In addition to this, environmental stress and social stress are thought to contribute to the development of these symptoms in menopausal women [4]. So far, hormone replacement therapy has been heavily used for the treatment of these symptoms for many years. However, hormone replacement therapy has potentially severe adverse consequences, including increased risk for coronary heart disease, cancer, stroke, and weight gain [9–11]. Thus, various other methods are also used to treat multiple physical and psychiatric symptoms of menopause, including antidepressants, minor tranquilizers, and several traditional Japanese Kampo medicines [12–15].

It is well known that kamishoyosan (KSS) is a traditional Japanese Kampo medicine that is widely used for the treatment of various neuropsychiatric symptoms in perimenopausal and postmenopausal women [12, 16, 17]. However, the molecular mechanisms underlying KSS-mediated attenuation of neuropsychological symptoms and stress-response behaviors in perimenopausal and postmenopausal women are unknown. Indicating one potential mechanism, major components of KSS, including Bupleuri Radix and Angelicae Radix, bind to multiple psychiatrically relevant receptors, such as the 5-HT1A receptor (5-HT1AR) [18, 19]. Previous studies have suggested that the 5-HT1AR plays an important role in both the pathogenesis and treatment of depressive disorders [20]. 5-HT1AR classically couples to an inhibitory G-protein that inhibits adenylyl cyclase, resulting in decreased cyclic adenosine monophosphate (cAMP) production and PKA activity [21]. The 5-HT1AR is highly expressed postsynaptically in the limbic regions, including the hippocampus, and in the frontal and entorhinal cortices [22, 23]. Several studies have reported that the hippocampal deficit of 5-HT1AR is associated with depressive disorders [20, 24, 25]. A recent study using a stress-induced psychiatric disorder animal model reported that decreased 5-HT1AR levels accompanied by altered cAMP-PKA-CREB signaling in the hippocampus are linked to the pathophysiological process of depressive disorders [26]. CREB signaling plays crucial roles in neurodevelopment, synaptic and neural plasticity, and neuroprotection [27]. Furthermore, chronic administration of antidepressants upregulates PKA activity and its downstream transcription factor CREB, resulting in the induction of CREB-dependent BDNF expression in the hippocampus [28]. Many studies have reported that PKA-CREB signaling is closely linked to depression and its treatment [29–32].

The male rodent model of depression-like signs, used in our studies, involves repeated water-immersion and restraint stress (WIRS) [33–35]. This induces hypothalamic-pituitary-adrenal (HPA) axis activation and reduces adult neurogenesis in the hippocampus, which are both well-known endophenotypes of depression [33]. Additionally, ovariectomized (OVX) female mice are a common rodent model of menopause [36, 37]. In the present study, we established a mouse model of postmenopausal depression by chronically exposing OVX mice to WIRS and investigated whether KSS treatment improves depression-like behaviors in this model. Furthermore, we examined the effects of KSS on 5-HT1AR levels and the PKA-CREB-BDNF signaling pathway in the hippocampus of this mouse model.

## 2. Materials and Methods

### 2.1. Ethical Approval

All animal care and handling procedures were approved by the International Animal Care and Use Committee of Kindai University (No. KAME-25-009), and the Guiding Principles for the Care and Use of Laboratory Animals and the United States National Institutes of Health Guide for the Care and Use of Laboratory Animals were closely adhered to.

### 2.2. Animals and Stress Exposure

C57BL/6 female mice were purchased from SLC (Japan SLC, Inc., Hamamatsu, Japan) at 10 weeks of age. Three mice per cage were housed in a 22 ± 2°C, 55 ± 10% humidity room on a 12-h light/dark cycle (lights on at 07:00 a.m. and off at 07:00 p.m.). Animals were fed standard laboratory food (CE-2, CLEA Japan, Inc., Tokyo, Japan) and water ad libitum.

All female mice were bilaterally ovariectomized (OVX) at 12 weeks of age and divided into the following three groups: an ovariectomized and nonstressed group (control OVX mice), an ovariectomized and chronically stressed group (OVX + stress mice), and an ovariectomized, chronically stressed, and KSS-treated group (OVX + stress + KSS mice). After 2 weeks of postoperative recovery, chronic stress exposure was performed, as previously described [33–35]. OVX mice were exposed to chronic WIRS for 3 weeks (from 14 weeks of age to 17 weeks of age). In brief, mice were placed in a 50 mL conical polypropylene centrifuge tube and immersed vertically to the level of the xiphoid process in a 23°C water bath for 2 h once daily for 3 weeks.

### 2.3. Drug Administration

KSS is composed of ten dried medicinal herbs in the following ratios: 13.3% Bupleuri Radix (*Bupleurum falcatum*), 13.3% Paeoniae Radix (*Paeonia lactiflora*), 13.3% Atractylodis Rhizoma (*Atractylodes ovate*), 13.3% Angelicae Radix (*Angelica acutiloba*), 13.3% Hoelen (*Poria cocos*), 8.9% Gardeniae Fructus (*Gardenia jasminoides*), 8.9% Moutan Cortex (*Paeonia suffruticosa*), 6.7% Glycyrrhizae Radix (*Glycyrrhiza uralensis*), 4.4% Zingiberis Rhizoma (*Zingiber officinale*), and 4.4% Menthae Herba (*Menthae arvensis*) [38]. KSS is produced by extracting these ten medical herbs with purified water at 95°C for 1 h, and the extraction solution is then separated from the insoluble waste and concentrated by removing water under reduced pressure. Spray-drying is used to produce a dried extract powder. The present study relied on dry powdered extracts of KSS supplied by Tsumura & Co. (Tokyo, Japan), and dry powdered extracts of KSS were mixed with CE-2 chow at a final concentration of 3%. Mice in the OVX + stress + KSS group were fed with this chow from a week before chronic stress exposure to the end of experimentation (from 13 weeks of age to 17 weeks of age).

### 2.4. Measurement of Plasma Corticosterone Levels

Plasma corticosterone levels were measured as previously described [33]. One day after the end of the 3-week chronic stress exposure period, mice were deeply anesthetized, and their blood was collected into heparin tubes between 11:00 a.m. and 01:00 p.m. These tubes were immediately placed on ice and then centrifuged at 1,000*g* for 15 min at 4°C. Plasma samples were stored at −80°C prior to conducting enzyme-linked immunoassays (ELISA). Plasma corticosterone levels were determined in duplicate using a corticosterone enzyme-linked immunoassay kit (Arbor Assays Inc., Arbor, MI, USA; K014), according to the manufacturer's instructions.

### 2.5. Forced Swim Test

The forced swim test (FST) was performed 2 days after the end of the 3-week chronic stress exposure period between 09:00 and 11:00 a.m., as previously described [33]. The apparatus for this test consisted of a single acrylic cylinder (25 cm height × 20 cm diameter) that was filled with 23°C water to a height of 11 cm. Mice were placed in these cylinders, and their time spent immobile was recorded for 6 min.

### 2.6. BrdU Injections and Immunohistochemistry

BrdU incorporation and immunostaining were conducted as previously described [33]. Mice were injected intraperitoneally with BrdU (Merck KGaA., Darmsfadt, Germany; Sigma-Aldrich; 150 mg/kg body weight) 3 days after the end of the 3-week exposure to chronic stress and transcardially perfused with 4% paraformaldehyde 2 h later. Their brains were removed and immersion-fixed in 4% paraformaldehyde at 4°C overnight. After fix, brains were suspended in 30% sucrose overnight at 4°C. Free-floating tissue sections that were 30 *μ*m thick were treated with 2 N HCl for 15 min at 37°C and incubated in a 0.1 M boric acid solution for 10 min at room temperature. After blocking with 5% bovine serum albumin (BSA) and 0.3% Triton X-100, anti-BrdU (Abcam plc., Cambridge, UK; ab6326) and anti-doublecortin (DCX) (Abcam plc., Cambridge, UK; ab18723) antibodies were applied at 1 : 400 dilutions overnight at 4°C. Secondary antibodies (Thermo Fisher Scientific Inc., Waltham, MA, USA) were then applied at 1 : 1000 dilutions for 2 h at room temperature. For visualization of nuclear DNA, the sections were stained with 1 *μ*g/mL DAPI (Thermo Fisher Scientific Inc.). The number of BrdU-positive (BrdU+) cells or BrdU- and DCX- positive (BrdU + DCX+) cells was counted on every 6th section in the brain region encompassing the entire rostrocaudal extent of the dentate gyrus. The number of BrdU + cells or BrdU + DCX + cells in the dentate gyrus was estimated by multiplying the counted cell number by six.

### 2.7. Quantitative Real-Time PCR

Total RNA was prepared from the hippocampi of mice with Isogen II (NipponGene, Toyama, Japan), according to the manufacturer's instructions. Reverse transcription of 1 *μ*g total RNA was performed using a High-Capacity cDNA Reverse Transcription Kit (Thermo Fisher Scientific Inc., Waltham, MA, USA). To evaluate the expression of *5-HT1AR*, *BDNF*, and *GAPDH*, quantitative real-time PCR (qRT-PCR) was conducted using the KOD SYBR qPCR Mix (TOYOBO Co., Ltd., Osaka, Japan). The following sets of forward/reverse primers were used: 5-HT1AR, 5′-GGATGTTTTCCTGTCC-TGGT-3′/5′-CACAAGGCCTTTCCAGAACT-3′; BDNF, 5′-CGCCATGCAATTTCCACTATCAATAATTTA-3′/5′-CGCCTTCATGCAACCGAAGTATG-3′; and GAPDH, 5′-GTGTTCCTACCCCCAATGTG-3′/5′-AGGAGACAAC-CTGGTCCTCA-3′. GAPDH was used as an internal housekeeping gene. Specific ratio comparisons (gene of interest/GAPDH) were used to assess differences in transcript expression between the groups.

### 2.8. Western Blot Analyses

Western blot analyses were performed as previously described [35]. We used the following primary antibodies: anti-5-HT1AR (Abcam plc., Cambridge, UK; ab 85615); anti-phospho-PKA (1 : 1000, Cell Signaling Technology, Danvers, MA, USA; Cat. # 4781); anti-CREB (1 : 1000, Cell Signaling Technology; Cat. # 9197); anti-phospho-CREB (1 : 1000, Cell Signaling Technology; Cat. # 9198); anti-BDNF (1 : 500, Abcam plc.; ab203573); and anti-GAPDH (1 : 500; Santa Cruz Biotechnology Inc., Dallas, TX, USA; sc-32233). To detect p-CREB and CREB, the membranes were blocked with 5% BSA and 0.1% Tween-20 in Tris-buffered solution (TBS) for 1 h at room temperature. Then, antigen-antibody reactions were performed using Can Get Signal solution (TOYOBO Co., Ltd., Osaka, Japan). Immunodetection was performed using the ECL Prime Western Blotting Detection System (GE Healthcare Systems Inc., Chicago, IL, USA) with horseradish peroxidase-conjugated secondary antibodies (1 : 5,000; Cell Signaling Technology Inc., Danvers, MA, USA). Densitometric quantification was performed using ImageJ software (National Institute of Health) with GAPDH as the loading control.

### 2.9. *In Situ* Hybridization

Murine 5-HT1AR cDNA fragments were obtained via PCR using the following primers: 5′-TCTATATTCCGCTGCTGCTC- 3′ and 5′-TTGAGTGAACAGGAAGGGTC- 3′, as described previously [39] and used as templates for probe synthesis. Digoxigenin-labeled RNA probe synthesis and hybridization procedures were performed as described previously [40]. Hippocampal 5-HT1AR mRNA intensities were quantified using ImageJ software.

### 2.10. Statistical Analyses

Data are expressed as mean± standard error of the mean (SEM), with the number of experiments indicated by (*n*). Tukey–Kramer's post hoc tests following analyses of variance (ANOVAs) were used for all multiple comparisons. *P* values <0.05 were considered statistically significant.

## 3. Results

### 3.1. KSS Normalized Stress-Upregulated Plasma Corticosterone Levels

**Figure 1:**
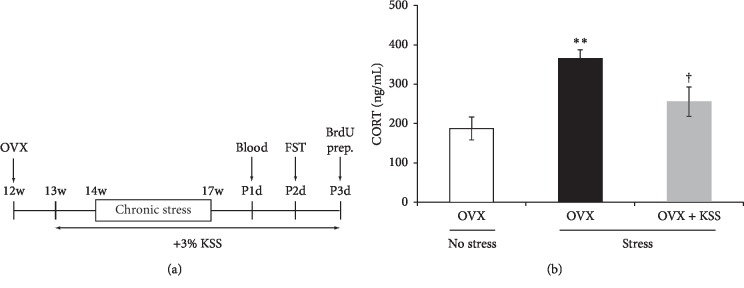
Effects of chronic stress and KSS treatment on plasma corticosterone levels in OVX mice. (a) Experimental design. (b) Plasma corticosterone levels were measured by ELISA using the blood samples collected one day after the end of the 3-week chronic stress exposure period. Results are shown as the means ± SEM (OVX group: *n* = 8; OVX + stress group: *n* = 10; OVX + stress + KSS group: *n* = 11). ^*∗∗*^*P* < 0.01 versus OVX group; ^†^*P* < 0.05 versus OVX + stress group by Tukey–Kramer posttest following one-way ANOVA. KSS, kamishoyosan; OVX, ovariectomized.

The HPA axis is continuously activated by chronic stress exposure and leads to upregulated plasma corticosterone levels [33, 41]. To verify whether the HPA axis is similarly activated in our mouse model of postmenopausal depression, we first measured plasma corticosterone levels in the OVX + stress group and found that plasma corticosterone levels increased compared to control OVX mice (Figures 1(a) and 1(b)). However, plasma corticosterone levels were significantly decreased in the OVX + stress + KSS mice compared with OVX + stress mice (Figure 1(b)). This suggests that KSS reduced HPA axis activation in OVX + stress mice.

### 3.2. KSS Reduced Stress-Induced Depression-Like Behavior

**Figure 2:**
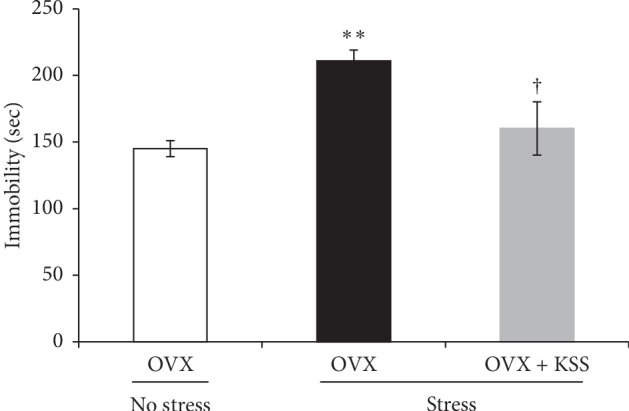
Effects of chronic stress and KSS treatment on OVX mouse depression-like behavior. Depressive behavior was assessed by the forced swim test. Immobility time was significantly increased by chronic stress, which was normalized by KSS administration. Results are shown as the means ± SEM (OVX group: *n* = 10; OVX + stress group: *n* = 10; OVX + stress + KSS group: *n* = 11). ^*∗∗*^*P* < 0.01 versus OVX group; ^†^*P* < 0.05 versus OVX + stress group by Tukey–Kramer posttest following one-way ANOVA. KSS, kamishoyosan; OVX, ovariectomized.

To evaluate postmenopausal depression-like behaviors in OVX + stress mice, we conducted a depression-related behavioral test (Figure 1(a)). In the FST, OVX + stress mice spent significantly more time immobile than control OVX mice, indicating that chronic stress increased depression-like signs in OVX mice (Figure 2). On the other hand, OVX + stress + KSS mice displayed immobility times comparable with those measured in control OVX mice (Figure 2). This suggests that KSS administration ameliorated chronic stress-induced depression-like behavior in these animals.

### 3.3. KSS Prevented a Stress-Induced Decrease of Neurogenesis in the Hippocampal Dentate Gyrus

**Figure 3:**
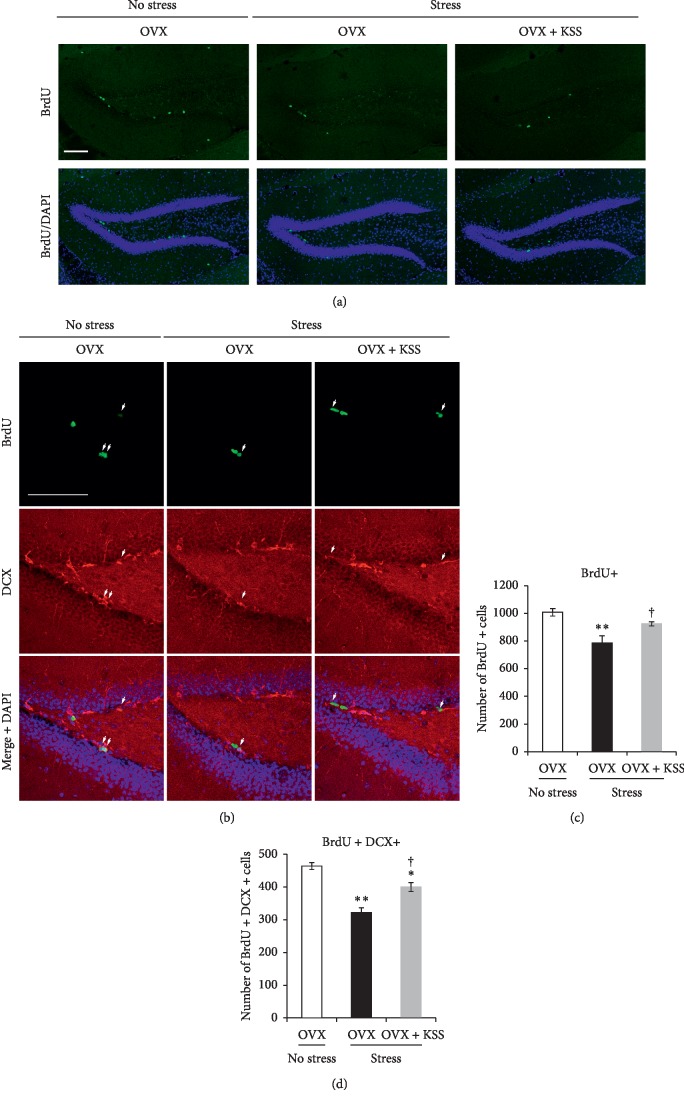
Effects of chronic stress and KSS treatment on adult neurogenesis in OVX mice. (a) Representative staining of BrdU (green) and DAPI (blue) in the hippocampal dentate gyrus. (b) Representative staining of BrdU (green), DCX (red), and DAPI (blue) in the hippocampal dentate gyrus. Arrowheads indicate colocalization. (c) The number of BrdU + cells in the hippocampal dentate gyrus is shown as the means ± SEM (*n* = 4). (d) The number of BrdU + DCX + cells in the hippocampal dentate gyrus is shown as the means ± SEM (*n* = 3). ^*∗*^*P* < 0.05 and ^*∗∗*^*P* < 0.01 versus OVX group; ^†^*P* < 0.05 versus OVX + stress group by Tukey–Kramer posttest following one-way ANOVA. Scale bar: 100 *μ*m.

Abnormal levels of adult neurogenesis in the hippocampal dentate gyrus have been robustly linked to depressive disorders [42, 43]. We previously reported that repeatedly exposing male mice to WIRS resulted in a significant decrease in neurogenesis in the dentate gyrus of the adult hippocampus [33]. Using an in vivo BrdU labeling assay to measure cell proliferation in the dentate gyrus (Figure 1(a)), we found that while the number of BrdU + cells were significantly decreased in OVX + stress mice compared with control OVX mice, BrdU + cell numbers were recovered in OVX + stress + KSS mice (Figures 3(a) and 3(c)). Furthermore, double immunohistochemical staining for BrdU and DCX to label newly generated neurons revealed that OVX + stress mice showed a significant reduction in the number of BrdU + DCX + cells as compared to that in control OVX mice, while BrdU + DCX + cell numbers were restored in OVX + stress + KSS mice (Figures 3(b) and 3(d)). These results suggest that KSS treatment normalized neurogenesis levels in the hippocampal dentate gyrus in OVX + stress mice.

### 3.4. KSS Normalized Decreased 5-HT1AR Expression Levels in the Hippocampus

**Figure 4:**
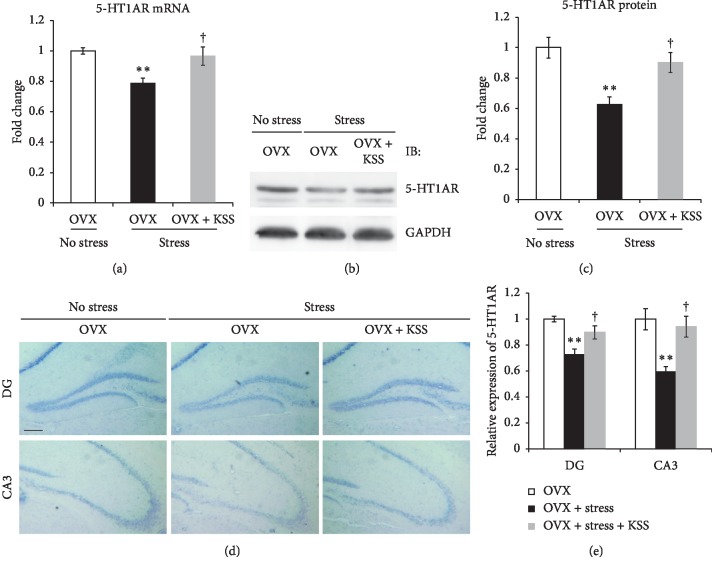
Effects of chronic stress and KSS treatment on hippocampal 5-HT1AR expression in OVX-mice. (a) Expression of *5-HT1AR* mRNA in the hippocampus was quantified via qRT-PCR. Results are shown as means ± SEM (*n* = 6). (b) 5-HT1AR expression level was assessed via western blot. (c) Densitometric quantification of western blot analyses of 5-HT1AR. Results are shown as means ± SEM (*n* = 3). (d) *In situ* hybridization analyses of brain sections from OVX mice (left panel), OVX + stress mice (middle panel), and OVX + stress + KSS mice (right panel) with antisense RNA probes to *5-HT1AR* mRNA. (e) Quantification of *5-HT1AR* signals in the DG and CA3. Results are shown as means ± SEM (*n* = 3). ^*∗∗*^*P* < 0.01 versus OVX group; ^†^*P* < 0.05 versus OVX + stress group by Tukey–Kramer posttest following one-way ANOVA. Scale bar: 200 *μ*m.

The above findings indicated that KSS exerted antidepressive effects on OVX + stress mice in the present study. Previous studies have reported that hippocampal deficits in the 5-HT1AR are associated with the pathogenesis of depressive disorders [26, 30]. Therefore, we examined the effects of chronic stress and KSS treatment on *5-HT1AR* mRNA expression levels in the hippocampus by qRT-PCR. As shown in Figure 4(a), *5-HT1AR* mRNA was significantly decreased in OVX + stress mice compared to that in control OVX mice. On the other hand, these levels were restored in OVX + stress + KSS mice (Figure 4(a)). We next assessed *5-HT1AR* mRNA localization in the hippocampus by *in situ* hybridization analysis. *5-HT1AR* mRNA expression was detected in granule cells of the dentate gyrus and pyramidal cells of the CA regions. *5-HT1AR* mRNA expression levels in the granule cells of OVX + stress mice were decreased as compared to those in controls (Figures 4(d) and 4(e)). On the other hand, *5-HT1AR* mRNA expression in these cells was enhanced in OVX + stress + KSS mice compared to OVX + stress mice (Figures 4(d) and 4(e)). Similarly, *5-HT1AR* expression in the CA3 area decreased in OVX + stress mice compared to control OVX mice (Figures 4(d) and 4(e)). These decreased *5-HT1AR* expression levels were restored in OVX + stress + KSS mice (Figures 4(d) and 4(e)). Furthermore, western blot analysis revealed that 5-HT1AR protein levels in the hippocampus decreased in OVX + stress mice compared to control OVX mice, while the expression level was recovered in OVX + stress + KSS mice (Figures 4(b) and 4(c)). These results suggest that KSS treatment normalized 5-HT1AR expression levels in the hippocampus in stressed OVX mice.

### 3.5. KSS Upregulated the Expression of p-PKA, p-CREB, and BDNF in the Hippocampus

**Figure 5:**
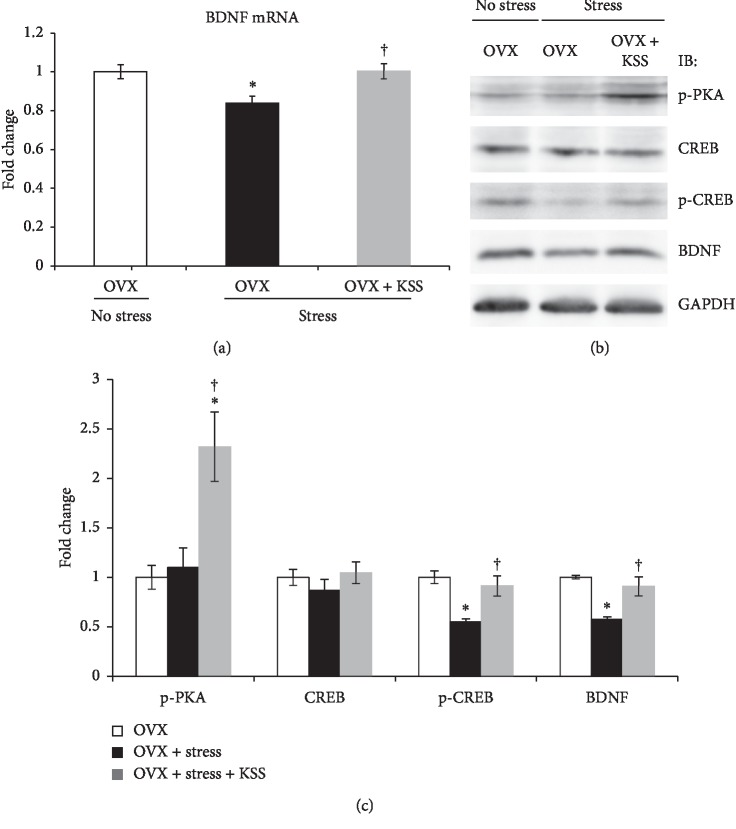
Effects of chronic stress and KSS treatment on PKA-CREB-BDNF signaling pathway in the hippocampus of OVX mice. (a) Expression of *BDNF* mRNA in the hippocampus was quantified via qRT-PCR. Results are shown as the means ± SEM (*n* = 7). (b) p-PKA, CREB, p-CREB, and BDNF protein levels were assessed via western blot. (c) Densitometric quantification of western blot analyses of p-PKA, CREB, p-CREB, and BDNF (*n* = 5-6). ^*∗*^*P* < 0.05 versus OVX group; ^†^*P* < 0.05 versus OVX + stress group by Tukey–Kramer posttest following one-way ANOVA.

Animal models of psychiatric disorders show decreased hippocampal 5-HT1AR levels accompanied by impaired cAMP-PKA-CREB signaling [26]. Therefore, to examine the effects of chronic stress and KSS treatment on this signaling pathway, we analyzed the expression level of PKA, CREB, and BDNF by qRT-PCR and western blot analysis. As shown in Figure 5(a), *BDNF* mRNA expression in OVX + stress mice was significantly reduced compared to that in control OVX mice but recovered with KSS treatment (Figure 5(a)). Western blot analysis confirmed that p-CREB and BDNF protein expression levels in the hippocampi of OVX + stress mice were significantly decreased compared to that in control OVX mice, while p-PKA, p-CREB, and BDNF protein expression levels in the hippocampi of OVX + stress + KSS mice were increased compared to that in OVX + stress mice (Figures 5(b) and 5(c)). There were no significant differences in CREB levels among the groups (Figures 5(b) and 5(c)).

Taken together, our findings indicate that KSS increased 5-HT1AR levels and PKA-CREB-BDNF signaling in the hippocampus and normalized postmenopausal depression-like symptoms.

## 4. Discussion

Perimenopausal and postmenopausal women often encounter serious social and/or psychological problems, such as changes to their family structure or community, healthcare, and occupation [44–46]. These environmental challenges are accompanied by hormonal changes, which are related to the quality of life in perimenopausal and postmenopausal women, and play important roles in the pathogenesis of depressive disorders [4, 6, 38, 47]. KSS has been widely used to treat neuropsychiatric diseases such as anxiety, insomnia, irritability, depression, and sleep disorders in perimenopausal and postmenopausal women [12, 16, 17, 48]. However, the molecular mechanisms underlying the use of KSS for various neuropsychiatric symptoms in perimenopause and postmenopause remain unclear. It was recently reported that KSS alters gamma-aminobutyric acid A/benzodiazepine (GABA_A_/BZP) receptor activity in the frontal cortex and hippocampus of male mice and thus may have an anxiolytic effect [49, 50]. Furthermore, prior work reported that KSS treatment restores neurogenesis in male rats exposed to chronic stress, suggesting that KSS has antidepressant-like effects [51]. In the present study, we established a mouse model of postmenopausal depression using OVX and WIRS and found that KSS ameliorated chronic stress-induced depression by upregulating 5-HT1AR and activating the PKA-CREB-BDNF signaling pathway in the hippocampus.

OVX rodents are the most ubiquitous animal model for the study of menopausal behaviors [36, 37]. Female rodents are known to respond to stress more readily than male rodents, and female OVX rats, which exhibit decreased plasma estradiol levels, have higher levels of basal corticosterone than male rats [52, 53]. While previous studies have reported that chronic restraint stress-exposed OVX rodents show higher serum corticosterone levels compared with nonstressed OVX rodents [54, 55], no prior investigations have assessed plasma corticosterone levels in chronic WIRS-exposed OVX mice (Figure 1(a)). Thus, to evaluate HPA axis activation in our mouse model of postmenopausal depression, we first assessed whether OVX mice exposed to chronic WIRS showed upregulated plasma corticosterone levels along with depression-like behaviors and whether continuous KSS treatment during chronic stress exposure improved these signs. As demonstrated in Figures 1 and 2, our results showed that chronic stress increased plasma corticosterone levels and prolonged the immobility time in the FST in OVX mice, whereas these observations were ameliorated by continuous KSS treatment. These results are in line with a previous study which shows administration of KSS normalizes plasma corticosterone levels in chronic constriction injury model rats [56]. Furthermore, an in vivo BrdU labeling assay, coupled with BrdU and DCX double immunohistochemistry, showed that KSS treatment prevented the chronic stress-induced decrease in neurogenesis in the hippocampal dentate gyrus of our model mice, consistent with the previous study in male rats exposed to chronic stress (Figure 3) [51]. Rodent studies suggest that decreased adult hippocampal neurogenesis is a sign of depression [33, 42, 43]. Taken together, these results indicate that KSS treatment ameliorated chronic stress-induced depressive signs in our model mice.

Next, we provided the first evidence that the antidepressive effects of KSS are accompanied by a recovery of 5-HT1AR expression in the hippocampus (Figure 4). We found that the 5-HT1AR expression level was significantly lower in the hippocampus of OVX mice after 3 weeks of chronic stress and was recovered by KSS treatment (Figure 4). Consistent with our data, recent studies using psychiatric animal models have demonstrated that antidepressive behaviors coincide with the restoration of hippocampal 5-HT1AR expression [26, 30]. Repeated exposure to stressful events is associated with an increased risk for depression [33, 35]. Repeated environmental stress activates the HPA axis, which results in increased plasma corticosterone levels [57]. Plasma corticosterone regulates the secretion of corticotropin-releasing factor (CRF) from the neuronal terminals of the paraventricular nucleus [57]. CRF activates the dorsal raphe nucleus and alters the serotonergic system, blunting 5-HT1AR and 5-HT2AR functioning [58, 59]. This CRF-induced 5-HT1AR and 5-HT2AR dysregulation occurs not only in the dorsal raphe but also in the cortex and hippocampus, areas closely associated with depressive disorders [60, 61]. In human studies, there is growing evidence supporting a deficiency of 5-HT1AR in the hippocampus of depressed patients and suicidal subjects [20, 24, 25]. Critically, chronic administration of corticosterone suppresses 5-HT1AR mRNA expression in the dentate gyrus of the hippocampus [62–64]. Our model mice exhibited elevated plasma corticosterone concentrations, which were ameliorated by KSS treatment (Figure 1(b)). Given this result and the prior work mentioned above, corticosterone downregulation by KSS treatment might be associated with the recovery of 5-HT1AR expression in the hippocampus. However, the molecular details of how KSS upregulates 5-HT1AR transcripts in the hippocampus remain unknown. The murine 5-HT1AR promoter region contains a novel type of negative glucocorticoid response element (nGRE) that suppresses 5-HT1AR mRNA transcription [65]. This nGRE mediates repression by glucocorticoid receptor (GR) and mineralocorticoid receptor (MR), which are highly expressed in the hippocampus and involved in the regulation of corticosterone release [57, 65]. Therefore, to understand the underlying molecular mechanism of KSS on 5-HT1AR upregulation, it is important to examine the effects of chronic WIRS and KSS treatment on hippocampal MR and GR expression levels.

We further demonstrated that KSS treatment significantly increased PKA-CREB-BDNF signaling in the hippocampus (Figure 5). Consistent with our data, recent study has reported that 5-HT1AR level and PKA-CREB-BDNF pathway in the hippocampus were downregulated in psychiatric model animals, whereas this downregulation was recovered by Chinese medicine [30]. The second messenger cAMP activates PKA, which in turn phosphorylates and activates CREB [28]. p-CREB induces BDNF transcription in the hippocampus, which is important for antidepressive effects [28]. A recent study reported that genetic deletion of the 5-HT1AR in mature granule cells of the hippocampal dentate gyrus impaired selective serotonin reuptake inhibitor- (SSRI-) induced antidepressive behaviors, neurogenesis, and BDNF expression, suggesting that the 5-HT1AR in mature granule cells is necessary for the expression of these antidepressant effects [66]. Therefore, determining whether and how KSS increases BDNF protein levels in mature granule cells will help us understand its antidepressant like-effects. Critically, Bupleuri Radix and Angelicae Radix, major components of KSS, are reported to have a binding affinity for 5-HT1AR [18, 19], and treatment with Bupleuri Radix extract significantly increased p-CREB and BDNF expression in cultured SH-SY5Y cells [67]. These studies suggest the possibility that the upregulation of p-CREB and BDNF expression after treatment with Bupleuri Radix extract might be because of 5-HT1AR modulation. Given this and previous studies, it is probable that KSS is not only involved in the recovery of 5-HT1AR expression but also in 5-HT1AR modulation, which might lead to upregulation of PKA-CREB-BDNF signaling in the hippocampus. However, CREB-BDNF signaling is regulated not only by 5-HT1AR but also by other receptors and by a variety of signaling cascades including Ras-MAPK and PI3K-Akt pathways, which are decreased by stress and depression [21, 28]. Additional mechanisms linking KSS to CREB-BDNF pathway activation are largely unknown. Future studies are needed to investigate the molecular details underlying the action of KSS on postmenopausal depression-like symptoms. Furthermore, it is important to determine which components of KSS are effective for postmenopausal depression using our models. Our results suggest that one or more active ingredients in KSS could be used as a possible alternative to current antidepressant drugs.

## 5. Conclusions

In summary, continuous treatment of postmenopausal depression-model mice with KSS led to antidepressant-effects via 5-HT1AR and the PKA-CREB-BDNF signaling pathway in the hippocampus. This is the first time that the molecular mechanisms underlying KSS's positive effects on postmenopausal depression have been directly assessed. These results may provide important clues about how KSS improves depression in postmenopausal women.

## Data Availability

The data used to support the findings of this study are available from the corresponding author upon request.
